# Identification of miRNAs involved in fruit ripening in Cavendish bananas by deep sequencing

**DOI:** 10.1186/s12864-015-1995-1

**Published:** 2015-10-13

**Authors:** Fangcheng Bi, Xiangchun Meng, Chao Ma, Ganjun Yi

**Affiliations:** Institute of Fruit Tree Research, Guangdong Academy of Agricultural Sciences, Guangzhou, 510640 China; Key Laboratory of South Subtropical Fruit Biology and Genetic Resource Utilization, Ministry of Agriculture, 510640 Guangzhou, China; Guangdong Provincial Key Laboratory of Tropical and Subtropical Fruit Tree Research, Guangzhou, 510640 China; Department of Postharvest Science of Fresh Produce, Agricultural Research Organization, The Volcani Center, Bet Dagan, 50250 Israel

**Keywords:** Musa acuminate, Fruit ripening, MiRNA, High-throughput sequencing

## Abstract

**Background:**

MicroRNAs (miRNAs) are a family of non-coding small RNAs that play an important regulatory role in various biological processes. Previous studies have reported that miRNAs are closely related to the ripening process in model plants. However, the miRNAs that are closely involved in the banana fruit ripening process remain unknown.

**Methods:**

Here, we investigated the miRNA populations from banana fruits in response to ethylene or 1-MCP treatment using a deep sequencing approach and bioinformatics analysis combined with quantitative RT-PCR validation.

**Results:**

A total of 125 known miRNAs and 26 novel miRNAs were identified from three libraries. MiRNA profiling of bananas in response to ethylene treatment compared with 1-MCP treatment showed differential expression of 82 miRNAs. Furthermore, the differentially expressed miRNAs were predicted to target a total of 815 target genes. Interestingly, some targets were annotated as transcription factors and other functional proteins closely involved in the development and the ripening process in other plant species. Analysis by qRT-PCR validated the contrasting expression patterns between several miRNAs and their target genes.

**Conclusions:**

The miRNAome of the banana fruit in response to ethylene or 1-MCP treatment were identified by high-throughput sequencing. A total of 82 differentially expressed miRNAs were found to be closely associated with the ripening process. The miRNA target genes encode transcription factors and other functional proteins, including SPL, APETALA2, EIN3, E3 ubiquitin ligase, β-galactosidase, and β-glucosidase. These findings provide valuable information for further functional research of the miRNAs involved in banana fruit ripening.

**Electronic supplementary material:**

The online version of this article (doi:10.1186/s12864-015-1995-1) contains supplementary material, which is available to authorized users.

## Background

Fruit ripening is a complex, genetically programmed processes involving marked changes in the color, flavor, aroma, texture, and nutritional content of the fruit [1, 2]. Many advances in our understanding of the genetic and molecular mechanisms involved in fruit ripening have been achieved in the model fruit tomato through the studies of various ripening-related mutants. These mutants include ripening inhibitor (*rin*), nonripening (*nor*), colourless nonripening (*Cnr*), green ripe (*Gr*), and never ripe and transcription factors such as TOMATOAGAMOUS-LIKE 1 (*TAGL1*), *AP2a*, ETHYLENE RESPONSE FACTOR 6 (*ERF6*) and FRUITFULL 1 (*FUL1*) [3]. Banana (*Musa acuminata*, AAA group), a typical climacteric fruit, is one of the most important fruit crops in tropical and subtropical countries with a high nutritional value for human health. It comprises an important part in the diet of millions of people around the world. Usually, bananas are harvested at 80 % plump stage, and ripening treatment is necessary before the fruit can be eaten. However, the climacteric characteristic of the banana fruit results in a very short shelf life at ambient temperature [4]. It is therefore important to clarify the molecular mechanisms of the ripening process. Some genes involved in the ethylene biosynthesis and perception pathways have been identified in the banana fruit, such as 1-aminocyclopropane-1-carboxylic acid (ACC) synthase, ACC oxidase, the ethylene receptor and the CTR1 orthologous genes [5, 6]. Recent reports show that several transcription factors exhibit regulatory roles during banana ripening, including MADS-box, ethylene insensitive 3-like (EIL3), NAC, ERF, LBD and BSD [7–13]. However, the other molecular pathways of fruit ripening regulation, such as the miRNA regulation pathways, are largely unknown.

MicroRNAs (miRNAs) are class of important non-coding regulatory small RNAs (20–24 nt) that mediate gene expression at post-transcriptional and translation levels by degrading target mRNAs or repressing gene translation [14]. Great advances have been made in understanding miRNA function in various plant species. Previous research has shown that miRNAs are involved in various metabolic and biological processes in plants, including development, signaling, abiotic stress and regulation of symbiotic relationships [15]. Recently, many studies have suggested that miRNAs play an important role in regulating development and ripening of fruit [3, 16–27]. For instance, Silva et al. (2014) experimentally reported that over-expression of an AtMIR156b precursor generated abnormal flower and fruit morphologies in tomatoes by affecting the expression of genes associated with meristem maintenance and the formation of new organs [21]. MiR156/157 and miR172 could affect the ripening process of tomatoes by modulating the known ripening regulators CNR and SIAP2a [27]. Currently, due to the development of high-throughput sequencing technology, thousands of miRNAs from different plant species have been identified and registered in the miRBase21.0 database (http://www.mirbase.org/).

In bananas, small RNAs (sRNAs) have been annotated based on the ‘Pahang’ A genome sequence similarities with known miRNAs in other species using bioinformatic methods [28, 29]. Moreover, recently, Wen et al. (2014) identified 180 pre-miRNAs and 314 mature miRNAs from triploid edible banana leaves with high-throughput sequencing methods [30]. However, the miRNAs closely involved in the banana fruit ripening process remain unknown. In this study, we investigated the miRNA profiles of banana fruits after ethylene and 1-MCP treatment using an Illumina HiSeq 2000 platform. The differentially expressed miRNAs involved in fruit ripening were identified, and the corresponding target genes were predicted. Subsequently, the potential functions of the differentially expressed miRNAs and their target genes were discussed. In addition, the expression patterns of several miRNAs and their target genes were examined by qRT-PCR methods. This is the first study to prove that miRNAs are widely involved in regulating the ethylene-induced ripening process of banana fruit. These data provide novel insights into the molecular mechanisms of banana fruit ripening.

## Results and discussion

### Small RNA revealed by high-throughput sequencing in the banana fruit

The plant hormone ethylene plays a key regulatory role in climacteric fruit ripening, which triggers marked changes in fruit flesh color, texture, flavor, and aroma [1]. 1-Methylcyclopropene (1-MCP), a receptor-binding antagonist of ethylene, blocks the effects of ethylene and is used commercially to slow fruit ripening [31, 32]. To identify miRNAs involved in the banana fruit ripening process. We treated the banana fruit with ethylene or 1-MCP. The color, firmness and ethylene production of banan fruit have remarkably changed in day 3, no apparent changes were observed in 1-MCP treated fruit (Additional file 1). Meanwhile, three sRNA libraries were constructed from banana fruit treated with ethylene or 1-MCP or before treatment (Table 1, Additional file 1) and then sequenced on an Illumina HiSeq2000, which produced 11,293,424 raw reads, 11,210,838 raw reads, and 10,645,104 raw reads, respectively. After removing the low-quality reads, the poly A/T/G/C reads, the adaptor reads and the N% > 10 % reads, an average of 96.55 % of the total reads remained. There were 10,789,352 clean reads, 10,885,475 clean reads and 10,330,271 clean reads for the control group, the ethylene treatment group, and the 1-MCP treatment group, respectively (Table 1). A total of 3,504,905 (control), 7,779,447 (ethylene treatment) and 2,253,332 (1-MCP treatment) sequences were successfully mapped to the banana reference genome (Table 2). There were an average of 147,499 reads identified as putative known miRNAs and an average of 12,182 identified as novel miRNAs (Table 2).

**Table 1:** The results of sRNA sequences from three banana libraries

Read types	Small libraries			Average
	Control	Ethylene	1-MCP	
Raw reads	11,293,424 (100 %)	11,210,838 (100 %)	10,645,104 (100 %)	11,049,789
N% > 10 % reads	3,622 (0.03 %)	4,834 (0.04 %)	2 (0.00 %)	2,819.333
Low quality reads	5,637 (0.05 %)	8,127 (0.07 %)	2,452 (0.02 %)	5,405.333
with ployA/T/G/C reads	6,011 (0.05 %)	12,314 (0.11 %)	2,458 (0.02 %)	6,927.667
Adaptor reads	488,802 (4.33 %)	300,088 (2.68 %)	309,921 (2.91 %)	366,270.3
Clean reads	10,789,352 (95.54 %)	10,885,475 (97.10 %)	10,330,271 (97.04 %)	10,668,366
Total bases (G)	0.565	0.561	0.532	0.552667

**Table 2:** Numbers of reads for each small RNA classification identified

Read types	Small libraries			Average
	Control	Ethylene	1-MCP	
Total	4,244,311 (100 %)	9,011,022 (100 %)	2,654,040 (100 %)	5,303,124
Sequences mapped to genome	3,504,905 (82.58 %)	7,779,447 (86.33 %)	2,253,332 (84.9 %)	4,512,561
Known_miRNA	84,534 (1.99 %)	278,076 (3.09 %)	79,887 (3.01 %)	147,499
Novel_miRNA	6,569 (0.15 %)	24,746 (0.27 %)	5,232 (0.20 %)	12,182
NAT	335,003 (7.89 %)	982,987 (10.91 %)	192,444 (7.25 %)	503,478
TAS	107 (0.00 %)	120 (0.00 %)	77 (0.00 %)	101
rRNA etc^a^	368,684 (8.69 %)	1,189,320 (13.20 %)	252,346 (9.51 %)	603,450
Repeat	195,636 (4.61 %)	389,021 (4.32 %)	95,972 (3.62 %)	226,876
Exon_sense	63,740 (1.50 %)	247,752 (2.75 %)	43,521 (1.64 %)	118,337
Exon_antisense	20,298 (0.48 %)	50,874 (0.56 %)	13,656 (0.51 %)	28,276
Intron_sense	62,933 (1.48 %)	131,964 (1.46 %)	31,316 (1.18 %)	75,404
Intron_antisense	58,311 (1.37 %)	124,767 (1.38 %)	31,022 (1.17 %)	71,366
Un_annotated	2,309,090 (54.4 %)	4,359,820 (48.38 %)	1,507,859 (56.81 %)	2,725,590

^a^rRNA/snRNA/snoRNA/tRNA considered; *TAS* trans-acting small interfering RNA, *NAT* natural antisense short interfering RNA

**Fig. 1:**
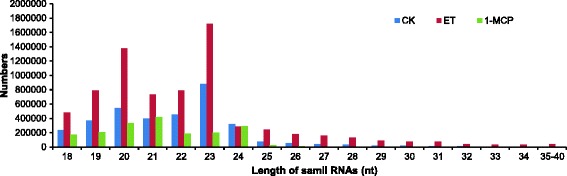
Length comparison of small RNAs from different treated banana fruit. CK, ET and 1-MCP stand for sample without any treatment; sample treated with ethylene and sample treated with 1-MCP (1-Methylcyclopropene) respectively. Y-axis represents the numbers of small RNA identified in this study. X-axis represents the length of small RNA. The length of the deep sequencing results were mainly between 18 and 24 nt. The number of 23 nt sequences is greater than the number of other sequence lengths in the control and the ET libraries. However, in the 1-MCP library, 21 nt sequences were the most abundant small RNAs

For further analysis, the number of small RNA sequences with 18–40 nt was counted (Fig. 1). In the control and ethylene treatment libraries, the 23 nt size class was the most abundant class of sRNAs, followed by the 20 nt, 22 nt, and 21 nt size class populations (Fig. 1). These results differ from those of *Medicago truncatula* [33], maize [34], potato [35], tomato [16], *Citrus trifoliate* [36], *Citrus sativus* [37], *Arabidopsis* [38], and rice [39], which all suggest that the most abundant small RNA length is 24 nt, and also differ from those of wheat, Chinese yew and grapevine [40–42], which demonstrated an sRNA length of 21 nt to be the most abundant. Moreover, in recent studies the enrichment of 22 nt was observed in the size distribution of sugarcane [43] and cucumber [44] sRNAs. This atypical situation of our data draws a parallel with the high number of 23 nt size class population in cucumber (*Cucumis sativus* L.) in response to cucumber green mottle mosaic virus Infection [44]. In addition, the miRNA results from bananas leaf indicated that the most abundant small RNA length is 21 nt, followed by 24 nt, 22 nt and 20 nt [30], implying a tissue-specific expression of small RNAs in banana species. Indeed, a recent report experimentally showed that the same miRNAs in different banana tissues exhibit differences in expression levels [29]. Recent study shows that 22 nt class sRNAs exhibit tissue-specific expression in sugarcane leaf tissue [43]. However, in the 1-MCP treatment libraries, the most abundant class of sRNAs is 21 nt sRNAs, followed by the 20 nt and 24 nt size class populations (Fig. 1). One possibility is that the sRNA populations could have been influenced under 1-MCP treatment in banana fruit.

Overall, these results suggest the existence of a specific, complex and diverse sRNA population and abiotic factors could affect the proportion of sRNAs of different length in the banana fruit. Moreover, except for the 24 nt sRNAs, all of the other examined sRNA classes exhibited strong induction after ethylene treatment, and showed a sharp reduction after 1-MCP treatment (Fig. 1), suggesting an important role for miRNA in the ethylene-induced ripening process of banana.

### Identification of known miRNAs in banana fruits

**Fig. 2:**
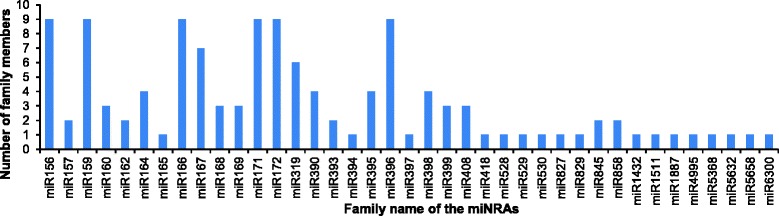
Conserved miRNAs and their family numbers in banana fruit. Y-axis represents the number of member in different miRNA family. Y-axis shows various conserved miRNA family identified in all three libraries. The miR156, miR159, miR166 miR171, miR172 and miR396 had nine members, however, 17 miRNA families had only one member

To identify the known miRNAs in banana fruits, small RNA sequences were mapped to the known mature plant miRNAs from the miRBase database. After Blastn searches and further sequence analysis, a total of 125 known miRNAs were identified (103, 112, and 94 in control, ethylene treatment and 1-MCP treatment, respectively), which belonged to 39 miRNA families (Additional file 2). Among these miRNAs, 80 known miRNAs (64 %) were detected in all three libraries, while 84 miRNAs were shared in at least two of three miRNAs libraries, implying a relatively stable component of miRNAs in banana fruit ripening (Additional file 2). In this study, 29 conserved miRNA families were confirmed (Table 3, Additional file 2); miR156, miR159, miR166, miR171, miR172, miR396 were the largest represented families with nine members, followed by miR167 and miR319, with seven and six members, respectively. Of the remaining 31 miRNA families, 14 comprised two to four members, and the others had only one member (Fig. 2; Additional file 2). In previous research, 32 potential miRNAs belong to 13 miRNA families were identified and validated using bioinformatic methods in banana [29]. With the exception of miR5538, all of the other previously identified miRNA families were found in this study. Similar to the tomato fruit [17], miR156, miR166 and miR171 were highly represented among the miRNA families. This differs from the Japanese apricot [19], pear [24], and peanut [45], which all found that miR156 was the largest miRNA family of the identified miRNA families. These results imply a conservation of miR156 across various species. In addition, 10 known miRNA sequences, belonging to 10 non-conserved miRNA families, including miR6300, miR5368, miR4995 and miR1511, were previously identified from one or few plants species and were expressed at very low levels (Table 3, Additional file 2).

**Table 3:** Summary information of known miRNA families and their transcript abundance identified in all libraries

miRNA family	No.of members	miRNA reads			Normalized reads			Fold Change		
		CK	ET	1-MCP	CK	ET	1-MCP	Log2 (ET/CK)	Log2 (1-MCP/CK)	Log2 (ET/1-MCP)
Conserved miRNA										
miR156	9	455	9460	147	107.20	1049.83	55.39	3.29	−0.95	4.24
miR157	2	16	1584	46	3.77	175.78	17.33	5.54	2.20	3.34
miR159	9	38786	103380	48416	9138.35	11472.62	18242.38	0.33	1.00	−0.67
miR160	3	28	111	80	6.60	12.32	30.14	0.90	2.19	−1.29
miR162	2	832	8566	398	196.03	950.61	149.96	2.28	−0.39	2.66
miR164	4	100	271	60	23.56	30.07	22.61	0.35	−0.06	0.41
miR165	1	5	70	6	1.18	7.77	2.26	2.72	0.94	1.78
miR166	9	2303	60599	1615	542.61	6724.99	608.51	3.63	0.17	3.47
miR167	7	997	4609	684	234.90	511.48	257.72	1.12	0.13	0.99
miR168	3	1743	10209	1448	410.67	1132.95	545.58	1.46	0.41	1.05
miR169	3	2	0	3	0.47	0.01	1.13	ND	ND	ND
miR171	9	22	90	6	5.18	9.99	2.26	0.95	−1.20	2.14
miR172	9	133	689	61	31.34	76.46	22.98	1.29	−0.45	1.73
miR319	6	84317	264863	69638	19865.89	29393.23	26238.49	0.57	0.40	0.16
miR390	4	8	34	7	1.88	3.77	2.64	1.00	0.48	0.52
miR393	2	304	1008	14	71.63	111.86	5.27	0.64	−3.76	4.41
miR394	1	38	45	26	8.95	4.99	9.80	−0.84	0.13	−0.97
miR395	4	62	66	50	14.61	7.32	18.84	−1.00	0.37	−1.36
miR396	9	13214	47358	9078	3113.34	5255.56	3420.45	0.76	0.14	0.62
miR397	1	1	0	0	0.24	0.01	0.01	ND	ND	ND
miR398	4	26	44	13	6.13	4.88	4.90	−0.33	−0.32	0.00
miR399	3	28	11	12	6.60	1.22	4.52	−2.43	−0.55	−1.89
miR408	3	27	80	26	6.36	8.88	9.80	0.48	0.62	−0.14
miR528	1	40	209	24	9.42	23.19	9.04	1.30	−0.06	1.36
miR529	1	964	2429	552	227.13	269.56	207.98	0.25	−0.13	0.37
miR530	1	0	0	1	0.01	0.1	0.38	ND	ND	ND
miR827	1	0	1	0	0.01	0.11	0.01	ND	ND	ND
miR858	2	62	58	60	14.61	6.44	22.61	−1.18	0.63	−1.81
miR845	2	1	0	2	0.24	0.01	0.75	ND	ND	ND
Non-conserved miRNA										
miR829	1	1	2	0	0.24	0.22	0.01	ND	ND	ND
miR418	1	0	1	0	0.01	0.11	0.01	ND	ND	ND
miR1432	1	0	1	0	0.01	0.11	0.01	ND	ND	ND
miR1511	1	3	3	2	0.71	0.33	0.75	ND	ND	ND
miR1887	1	1	0	0	0.24	0.01	0.01	ND	ND	ND
miR4995	1	11	8	9	2.59	0.89	3.39	−1.55	0.39	−1.93
miR5368	1	27	63	22	6.36	6.99	8.29	0.14	0.38	−0.25
miR5632	1	1	0	0	0.24	0.01	0.01	ND	ND	ND
miR5658	1	1	2	0	0.24	0.22	0.01	ND	ND	ND
miR6300	1	94	94	115	22.15	10.43	43.33	−1.09	0.97	−2.05

Normalized reads formula: normalized read count = (actual miRNA count / total count of clean reads) × 1,000,000. The clean reads in CK, ET and 1-MCP library are 4,244,311, 9,011,022 and 2,654,040 respectively (Table 2). After normalization, if the normalized read count of a given miRNA is zero, the expression value is set to 0.01 for further analysis, and if the read count of a given miRNA was less than five in both libraries, differential expression analysis was not performed owing to their too low expression level and replaced it with ND

Among the conserved miRNA families (Table 3), miR162, miR166, miR167, miR168 and miR396 were expressed at relatively high levels in all three libraries, which was consistent with previous reports from the tomato fruit [17]. These results suggested miRNAs are potentially important regulators of the fruit ripening process. Moreover, when the expression levels of known miRNA families under different treatments were analyzed, the expression levels of several miRNA families, including miR156, miR162, miR171, miR393 and miR172, showed high induction after ethylene treatment and inhibition after 1-MCP treatment (Fig. 2, Table 3). These effects imply an important role of these miRNA families during the banana fruit ripening process. In addition, various members within the same family showed variable expression levels. For instance, in the ethylene treatment library, the number of miR159 family member reads ranged from 1–65863 (Additional file 2), and the miR156 family (2–6422 reads) and the miR396 family (5–17955 reads) also exhibited variable read numbers. The expression patterns of the same miRNA family member within three libraries showed an increase after ethylene treatment and a decrease after 1-MCP treatment, suggesting the important regulatory role of these miRNAs in the ethylene-induced ripening process of banana (Additional file 2).

### Identification of novel miRNAs in the banana fruit

Based on the plant miRNA annotation criteria [46], the formation of a stable hairpin structure is the primary criterion of an miRNA. In total, 26 potential novel miRNAs were predicted from the three libraries (Additional file 3). In this study, the length of these 26 potential novel miRNA precursors ranged from 46–291 nt, which was close to the length of miRNA precursors in radishes (*Raphanus sativus* L.) [47]. The stem loop structures of these predicted miRNA precursors are shown in Additional file 4. Except for mac-nmiR6 and mac-nmiR14, the minimum free energy (MFE) folding value of these miRNA precursors ranged from −22.6 kcal/mol to −171.3 kcal/mol, with an average of 46.5 kcal/mol, which was consistent with the previously published low MFE characteristics of miRNA [48] and similar to the published sequencing data for pear miRNA precursors (51.48 kcal/mol) [24] and radish miRNA precursors (40.1 kcal/mol) [47]. Fifteen of the 26 novel miRNAs candidates were shared by all three libraries, and 25 predicted miRNAs candidates existed in both the control and the ethylene treatment libraries, but only 16 out of the 26 novel miRNA candidates existed in the 1-MCP treatment library. In addition, Meyers et al. (2008) reported the existence of complementary sequences increases the authenticity of predicted novel miRNAs [46]. In our study, 11 of the 26 novel miRNAs have complementary miRNAs. Moreover, all of the novel complementary miRNAs exhibited lower expression levels than the corresponding mature miRNAs (Additional file 3), which might be due to the rapid degradation of complementary strands during the biogenesis of mature miRNAs [38].

### Differentially expressed miRNAs of banana in response to ethylene or 1-MCP treatment

**Fig. 3:**
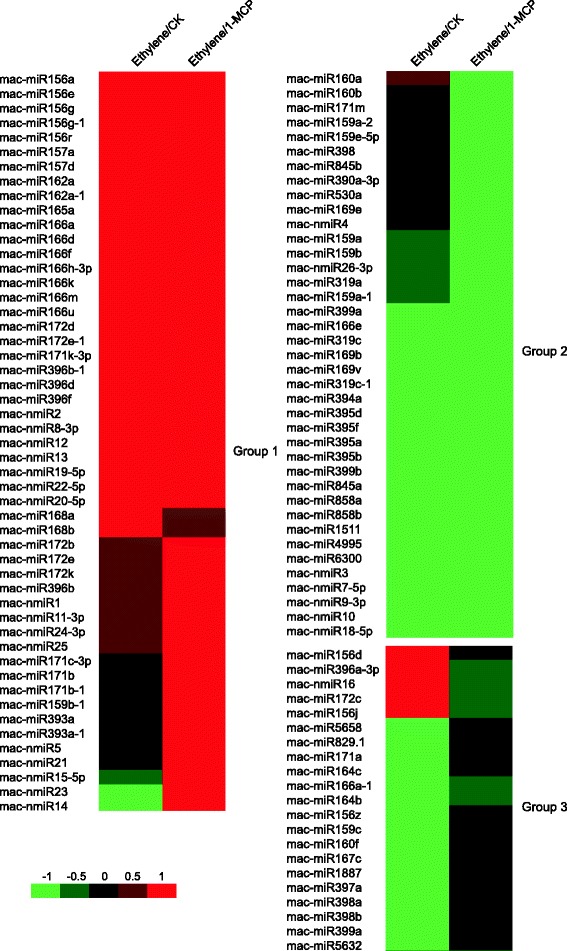
Differentially expressed miRNAs under two comparisons (ET/1-MCP, and ET/CK) in banana fruit. The bar represents the scale of relative miRNA expression (Log2 Fold change). The bottom left shows the color bar. The heatmap was generated by Heml 1.0

**Fig. 4:**
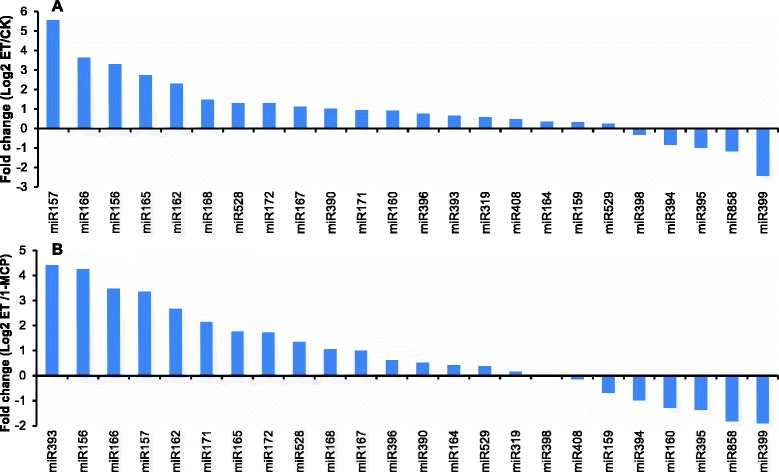
A comparison of the relative expression of differentially expressed conserved miRNA families. Comparison of ET treatment and CK (**a**), and comparison of ET treatment and 1-MCP treatment (**b**)

To determine the relationship between ethylene and miRNAs expression, exogenous ethylene and 1-MCP (a competitive inhibitor of ethylene action) were used to treat mature green fruit. Expression analysis was performed to identify differentially expressed miRNAs of the banana fruit response to ethylene or 1-MCP after three days of storage. Based on the selected criteria (at least one comparison has a fold change log_2_ scale value ≥ 1.0 or ≤ −1.0 with a *q*-value < 0.05), 87 known miRNAs and 24 novel miRNAs were identified as differentially expressed miRNAs (Additional file 2 and Additional file 3). Two comparisons were analyzed, ET/1-MCP, and ET/CK. The differentially expressed miRNAs were divided into three groups according to their expression patterns (Fig. 3 Additional file 5). The results show that 30 miRNAs were up-regulated in both comparisons, and the expression of 37 miRNAs was increased after ethylene treatment, including mac-miR172d, mac-miR172e-1, and mac-miR171k-3p. When compared with the 1-MCP treatment library, 49 miRNAs were up-regulated in the ethylene treatment library, suggesting that the 1-MCP ethylene antagonist affects the expression of some miRNAs. In contrast, 20 miRNAs were down-regulated in both comparisons. The expression of 32 miRNAs was decreased after ethylene treatment, including mac-miR156z. When compared with the 1-MCP treatment library, 33 miRNAs were down-regulated in the ethylene treatment library (Fig. 3). Among the 29 conserved miRNA families, 10 and three miRNA families were either up- or down-regulated, respectively, after ethylene treatment when compared with the control group (Fig. 4a). When compared with the 1-MCP treatment, 10 and four miRNA families were up-and down-regulated after ethylene treatment, respectively (Fig. 4b). These results indicate that these miRNAs might play important regulatory roles in ethylene-induced ripening of the banana fruit. In addition, previous studies have shown that several known miRNA families, including miR390, miR160, and miR171, were up-regulated after ethylene treatment in the tomato fruit [3], which is consistent with the results of this study (Fig. 4, Table 3). Moreover, several miRNA families (miR156 and miR172) were reported to be down-regulated in tomatoes after 6 h of ethylene treatment [3], while in this study, these miRNAs exhibited increased expression after 3 days of ethylene treatment in bananas. These differences might be because these miRNAs exhibit a dynamic expression in different time-points after ethylene treatment.

### Identification and classification of differentially expressed miRNAs targets

**Fig. 5:**
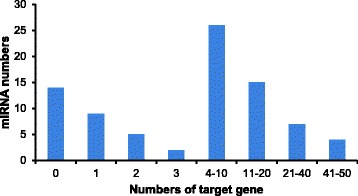
The distribution of target gene numbers for differentially expressed miRNAs among banana ripening process. The differentially expressed miRNAs of ethylene/1-MCP and their targeted genes are analyzed in this figure. The X-axis represents the interval for the target gene number of each miRNA. The Y-axis represents the number of miRNA in each interval

Most miRNA targets that are conserved in different plant species also have miRNA families with multiple target sites, suggesting that these miRNAs are functionally divergent. To further clarify the biological functions of the differentially expressed miRNAs in ethylene-induced ripening of bananas, we analyzed the target genes of 82 miRNAs with differential expression after ethylene treatment when compared with 1-MCP treatment. A total of 815 target genes for the 82 miRNAs were predicted. Among them, 534 and 55 target genes were predicted for 33 known and five novel up-regulated miRNAs, respectively (Additional file 6A), and 196 and 30 target genes were predicted for 27 known and three novel down-regulated miRNAs, respectively (Additional file 6B). Within these results, a single miRNA targeted multiple genes and multiple miRNAs regulated a single gene, suggesting the functional divergence of these miRNAs. As shown in Fig. 5, 26 differentially expressed miRNAs targeted four to 10 genes. Eight miRNAs had only one target gene, which may suggest unique regulatory functions. Meanwhile, we found that four miRNAs targeted to 40–50 target genes, indicating a wide regulatory function for these miRNAs. In addition, there were 14 differentially expressed miRNAs without a predicted target gene (Additional file 6), making it difficult to understand the function of these miRNAs. This is also indicative of the limitation of the current mapping methods. Further experiments and analyses are needed to confirm accurate functions of these miRNAs and their target genes.

The ripening-related targets for conserved miRNAs found in this study were similar to verified plant miRNA targets previously reported to have functional relevance. Some target genes were annotated as transcription factors (TFs). For example, the miR156 and miR157 family members were identified to target the squamosa promoter-binding-like protein genes (*SPLs*) [49, 50]. The miR172 family members were identified to target the floral homeotic protein APETALA 2 gene (*AP2*) [25]. The miR858 family members were identified to target the MYB family of transcription factors. Previous studies have proven that ectopic expression of strawberry *FaMYB1* could suppress anthocyanin and flavonol accumulation in tobacco [51]. The targets of the miR159 and miR319 family members belonged to the GAMYB transcription factor family, which is reported to affect flower development in the anther [52]. The miR169 family members were identified to a target nuclear transcription factor Y subunit A-9, which acts to control primary root growth in *Arabidopsis* [53]. Auxin response factor (ARF), a transcription factor, was found to regulate root growth in *Arabidopsis* [54, 55]. In our study, mac-miR160a and mac-miR160b potentially target ARF17, ARF18 and ARF22. Notably, the miR395 family members were identified to target ETHYLENE INSENSITIVE 3 (EIN3), a well-known transcription factor involved in the fruit ripening pathway [56]. Moreover, some target genes of differentially expressed miRNAs were annotated as functional proteins involved in fruit ripening. For instance, the miR160 family members were also identified to target an E3 ubiquitin ligase, an important component of the 26S proteasome. Recent studies indicated that the E2 enzyme, one component of the 26S proteasome, was involved in regulating fruit ripening [57] and that the 26S proteasome pathway also could affect the timing of fruit ripening through controlling the degradation of ethylene receptors in tomatoes [58]. Protein phosphatase 2A (PP2A) was reported to control ethylene synthesis by modulating the turnover of ACC synthase [59]. In this study, the protein phosphatase 2A regulatory B subunit gene (PP2A-B) was an identified target of several miR156 family members and mac-nmiR3. Interestingly, one of the targets for the novel miRNA mac-nmiR3 is β-galactosidase, which is a key softening-related enzyme [60]. The endo-1,3-beta-glucosidase targeted by mac-miR166u, mac-miR166e and mac-miR171b are also thought to be involved in fruit ripening [61, 62]. Additionally, some target genes were annotated as uncharacterized and hypothetical proteins. These results suggest that these miRNAs and their target genes might play crucial regulatory roles in the ethylene-induced ripening of bananas, but more experimental validation is needed to confirm their function and regulation.

**Fig. 6:**
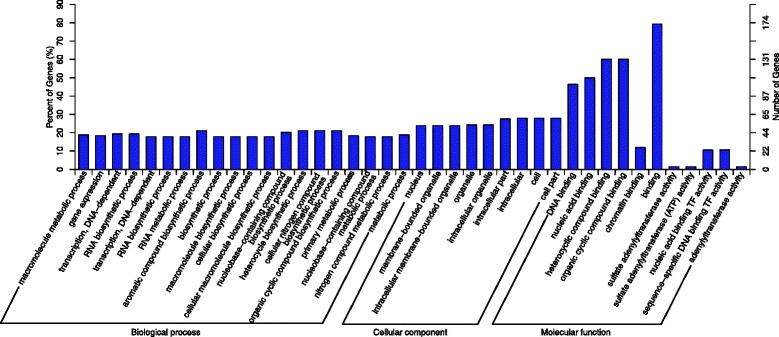
Gene ontology of the predicted targets for differentially expressed miRNAs. The targeted genes of differentially expressed miRNAs in the comparison of ethylene/1-MCP is analyzed in this figure. The right-hand-side scale is the targeted gene numbers corresponding to the GO terms. The left-hand-side scale is the percent of the targeted gene numbers corresponding to the GO terms

**Fig. 7:**
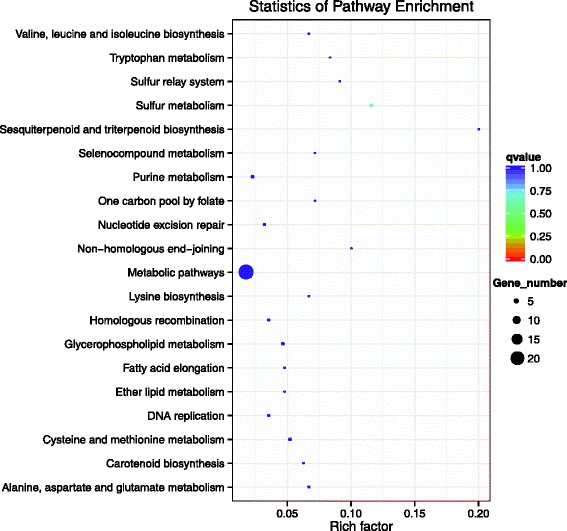
KEGG analysis of the 20 most enriched pathways. The coloring of the *q*-values indicates the significance of the rich factor. The circle indicates the target genes that are involved, and the size is proportional to the gene numbers. The x-axis represents name of enrichment pathway. The Y-axis represents rich factor

To further assess the putative functions of the 815 predicted targeted genes, a Gene Ontology (GO) analysis was performed using the Blast 2 GO program (http://www.blast2go.com) (Additional file 7). GO analysis contained three ontologies, biological processes, cellular components and molecular function. Twenty different biological processes, nine different cellular components and 11 different molecular functions were predicted (Fig. 6). The enrichment of each GO term within the biological processes and the cellular components were comparative, and most are involved in metabolic or biosynthetic processes. As for molecular functions, the binding terms and related binding terms comprised most of the targeted genes, which is consistent with a regulatory role for these miRNAs in the transcription and translation processes [63]. In addition, KEGG pathway analysis was performed to illuminate the biological interpretation of the differentially expressed miRNA target genes. The top 20 enriched pathways were identified with 53 genes (Fig. 7). “Metabolic pathways” was the most significantly enriched term with respect to the richness factor and gene number (22 genes) (Additional file 8), suggesting an important role for metabolism in the ethylene-induced ripening process of the banana fruit.

### Validation of the expression profiles of differentially expressed miRNAs and their target genes

**Fig. 8:**
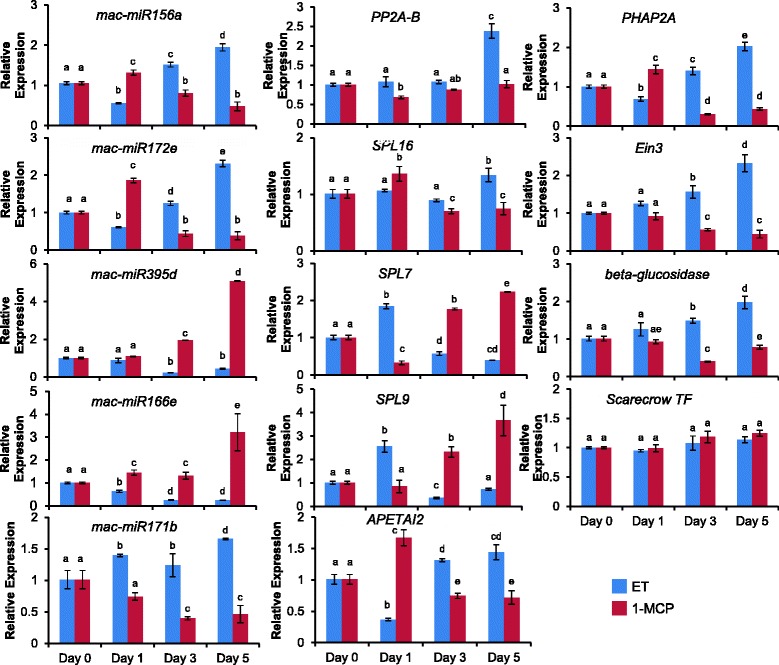
Quantitative expression analyses of several differentially expressed miRNAs and their target genes. The expression level in day 0 was set as 1. U6 rRNA and CAC (*clathrin adaptor complexes gene*) gene was used as the internal control for miRNA expression and targeted genes expression respectively. Each bar indicates the mean ± SE of triplicate assays. The different letters indicate significant differences at *P* < 0.05 using Fisher’s protected least significant difference (PLSD) tests

In this study, qRT-PCR was adopted to confirm the sequencing results of the differentially expressed miRNAs and their potential targets. Five differentially expressed miRNAs and nine corresponding targets were selected and validated with qRT-PCR. After ethylene treatment, the expression of mac-miR156a and mac-miR172e was down-regulated on day 1 and then up-regulated at day 3 and day 5 compared with that of day 0, while after 1-MCP treatment, the expression of these miRNA was up-regulated at day 1 and then down-regulated at days 3 and 5 compared with that of day 0 (Fig. 8). The expression of mac-miR395d and mac-miR166e were down-regulated in response to ethylene treatment and at similar levels or up-regulated in response to 1-MCP treatment. In addition, the expression levels of mac-miR171b were slightly increased after ethylene treatment and decreased in response to 1-MCP treatment. Overall, the results were consistent with the sRNA high-throughput sequencing data. Usually, miRNAs and their target genes have contrasting expression patterns. Thus, we also checked the expression levels of four predicted target genes (*SPL7, SPL9, SPL16, PP2A-B-ι*) for mac-miR156a, two predicted target genes (*APETALA2, PHAP2A*) for mac-miR172e, and one predicted target gene each (*Ein3, Scrarecrow TF, endo-1,3-beta-glucosidase 14*) for mac-miR395d, mac-miR171b and mac-miR166e, respectively. The results show the expected inverse relationship during ripening between mac-miR156a and its target genes *SPL7* and *SPL9,* mac-miR395d and its target gene *EIN3,* and mac-miR166e and its target gene *endo-1,3-beta-glucosidase 14* (Fig. 8). These data suggest that these miRNAs might affect ripening by regulating the expression of these potential fruit ripening-related genes. However, the expression of mac-miR171b and its target gene *Scrarecrow TF*, mac-miR156a and its target genes *SPL16* and *PP2A-B-ι,* mac-miR172e and its target genes *APETLA2* and *PHAP2A* show no contrasting expression patterns, indicating that these may not be target genes. This might be due to false prediction results from the highly repetitive motifs that correspond to the miRNA sequence in the gene sequences. Therefore, more experimental verification is needed to clarify the regulatory mechanism of miRNAs and their targets in the ripening process of the banana fruit.

Notablely, EIN3, the predicted target gene of miR395, a very important regulator in tomato ripening process. EIN3 can activate a variety of ethylene-response genes that involved in ripening [64]. Recent studies shown that RNAi-line of EIN3 displayed a delayed ripening phenotype, and exhibited normal vegetative growth in tomato [65, 66]. Moreover, according to our miRNAs sequencing results of different development stage fruit (data not shown), the expression level of miR395 in early mature green stage (30 days after flowering) shows about 3 times higher expression than that in late mature green stage (90 days after flowering). These results indicate that except regulating sulfate assimilation [67] miRNA395 maybe play important role in banana fruit ripening. Therefore, maybe we can delay banana fruit ripening process by overexpressing miRNA395.

## Conclusions

We identified a total of 125 known miRNAs and 26 novel miRNAs from the banana fruit in response to ethylene or 1-MCP treatment using Illumina Hiseq2000 sequencing methods. Analysis of differential expression data suggested these miRNAs play an important role in ethylene-induced ripening of bananas. In total, 815 corresponding target genes were predicted for these mRNAs, and their functions were discussed. GO enrichment analysis of target genes for the differentially expressed miRNAs revealed that some miRNA-targeted genes are closely related to fruit ripening. When combined with computational analysis and experimental confirmation, the findings of this study provide valuable information for further functional analysis of the miRNAs involved in banana fruit ripening.

## Methods

### Plant materials and sample collection

Banana fruit (*Musa acuminata*, AAA group, cv. Carvendish) at the 75–80 % plump stage were harvested from the plantation of the Institute of Fruit Tree Research, Guangdong Academy of Agricultural Science. The hands were separated into individual fingers and rinsed completely in water. The fruits were surface sterilized by dipping the samples into a 1 % sodium hypochlorite solution for 1 min and then immersing the samples in banana preservative reagent (GENGREEN, Zhuhai, China) for 3 min to prevent fungal disease. They were then allowed to dry at room temperature for 4 h before treatment. The fruits were treated with 100 μl/L of ethylene, 0.5 μl/L of 1-MCP or without treatment for 16 h in a closed chamber, and then stored at 22 °C. Samples were taken at 0, 1, 3, and 5 days. All samples were completely snap-frozen in liquid nitrogen and stored at −80 °C for further use. A sample before treatment was analyzed as the control. The samples before treatment and samples stored for 3 days after ethylene or 1-MCP treatment were used for high-throughput sequencing.

### Measurements of firmness and ethylene production rates

Fruit firmness was measured at the middle cross section of the fruit with a firmness tester GY-1. Six fruit fingers were measured at each sampling time. For assays of ethylene production rates, the fruit (3 fruit fingers) were sealed in plastic jars (2 L, 3 repeat jars) for 2 h at the sampling day, and then ethylene concentration was determined with portable ethylene analyzer (Absoger, EASL-1, France).

### RNA extraction and deep sequencing

Total RNA was extracted from the banana fruit using the hot borate method for isolation [68]. Three libraries were constructed and sequenced by Novogene (Beijing, China) using the Illumina HiSeq-2000 platform. For each sample, 3 μg of total RNA was used as the input material for the small RNA library. Sequencing libraries were generated using NEBNext® Multiplex Small RNA Library Prep Set for Illumina® (NEB, USA) according to the manufacturer’s recommendations, and index codes were added to attribute sequences to each sample. Briefly, the small RNAs were ligated with 3’ and 5’ adapters using T4 RNA ligase. The RNAs were subsequently transcribed to single-stranded cDNA using M-MuLV Reverse Transcriptase (RNase H^−^). Thereafter, PCR amplification was performed using LongAmp Taq 2X Master Mix and primers that anneal to adapters. PCR products were purified on an 8 % polyacrylamide gel (100 V, 80 min). After quality assessment using DNA High Sensitivity Chips, DNA fragments 140–160 bp in length were recovered and dissolved in 8 μl of elution buffer for sequencing.

### Bioinformatic analysis of sequencing data

After sequencing, clean reads were obtained by removing reads containing the following: poly-N, poly A/T/G/C, adapter-contaminated tags, low-quality reads and reads less than 18 nt in length from the raw data. All of the high quality sequences were considered significant and further analyzed. The small RNA tags were mapped to a reference sequence by Bowtie (http://banana-genome.cirad.fr/content/download-dh-pahang) [69]. The sequences matching non-coding RNAs (rRNA, scRNA, snoRNA, snRNA and tRNA) deposited in Rfam 10.1 database were removed from the sequences. The left unique sequences that mapped to known mature plant miRNAs in miRBase 21 (http://www.mirbase.org/) were considered to be known miRNAs. The remaining unannotated sRNA sequences were analyzed by an integrated combination of miREvo [70] and mirdeep2 [71] software to predict potential novel miRNAs through exploring hairpin structure, Dicer cleavage sites and the minimum free energy. The criteria used for novel miRNA were based on the work of Meyers et al. (2008) [46].

### Differential expression analysis of miRNAs in three libraries

The clean reads from each miRNA were normalized using the following formula: Normalized expression = mapped readcount/Total reads*1000000. After normalization, the miRNA expression profiles among three sRNA libraries (control, ethylene treatment and 1-MCP treatment) were compared. If the normalized read count of a given miRNA is zero, the expression value is set to 0.01 for further analysis. Differential expression analysis of two samples was performed using the DEGseq (2010) R package [72]. The *P-value* was adjusted using the *q*-value [73], and the *q-value* was set as the threshold for default significantly different expression. The differentially expressed miRNAs were screened with a threshold of fold change ≥ 1.0 or ≤ −1.0 (the log2 treatment/control) and with a *q-value* < 0.05. The target genes of each miRNA were predicted by aligning the miRNA sequence with the reference genome using psRobot_tar in psRobot1.2 [74] with default parameters. The GOSeq/topGO2.12 software and the KOBAS 2.0 software were used to annotate the functions of the predicted target sequences.

### qRT-PCR validation of differentially expressed miRNAs and their potential targets

Total RNA was extracted from the samples using TransZol plant solution (Transgen Biotech, Beijing, China) and then treated with RNase-free DNaseI. For each examined miRNA, 1 μg of DNase I-treated total RNA was used in a reverse transcription reaction with the Prime-Script® RT reagent Kit (Takara, Dalian, China), and U6 was used as the internal control. Reverse transcription was performed with the following conditions: 42 °C for 15 min, 85 °C for 5 s, and then held at 4 °C. For target genes, 1 μg of DNaseI-treated total RNA was used for synthesis with the Prime-Script® RT reagent Kit (Takara, Dalian, China) and an oligo (dT)_18_ primer, and the CAC (clathrin adaptor complexes gene) gene was used as an internal control [75]. Reverse transcription was performed using the following conditions: 37 °C for 15 min, 85 °C for 5 s, and then held at 4 °C. The cDNA was quantified by an LightCycler 480 Real-Time PCR system (Roche) using a 10-μl reaction mixture, which consisted of 3.4 μl of diluted cDNA, 1.6 μl of forward and reverse primer mix (2.5 μM of each primer), and 5 μl of 2X LightCycler480 SYBR Green I Master Mix (Roche). The following procedure was used for PCR amplification: 3 min at 95 °C for polymerase activation and then 45 cycles of 10 s at 95 °C, 15 s at 60 °C and 10 s at 72 °C. A melting curve analysis was performed to determine the specificity of the products. The expression level of the miRNAs and their target genes was quantified using the comparative 2^-ΔΔCt^ method [76]. The primer sequences were designed using Beacon Designer 7.0 software and are listed in Additional file 9. Fisher’s protected least significant difference (PLSD) test was used to assess any statistically significant differences in the results.

## Availability of supporting data

Sequencing data set supporting the results of this article is available in the repository of NCBI with the accession number PRJNA295255.

## Additional files

Additional file 1:
**Physiological changes of banana fruit after ethylene or 1-MCP treatment.** (DOCX 422 kb) Additional file 2:
**Detailed information on the known miRNAs.** MiRNA family, miRNA ID, location, length, sequence, total reads for mature miRNA are listed. (XLSX 1247 kb) Additional file 3:
**Detailed information on the identified novel miRNAs.** Novel miRNAs identified in three libraries. miRNA ID, length, sequence, location, MFE for precursor miRNA, structure of precursor, and total reads for mature miRNA are listed (XLSX 1228 kb) Additional file 4:
**The secondary structure of an identified novel miRNA precursor Secondary structure of 26 novel miRNA precursor is included.** (ZIP 95 kb) Additional file 5:
**The expression data of known miRNAs.** The detailed data of two comparison (ET/1-MCP, ET/CK). (ODS 22 kb) Additional file 6:
**The predicted target genes of differentially expressed miRNAs.** The target genes of up-regulated miRNAs (A) and the target genes of down-regulated miRNAs (B) in the comparison ET/CK. (ODS 27 kb) Additional file 7:
**Significant GO terms for target genes of differentially expressed miRNAs.** A total of 601 GO terms of the target genes annotated in this study. (XLSX 70 kb) Additional file 8:
**The most enriched pathways that were identified for the target genes.** A total of 53 most enriched pathways of target gene annotated in this study. (XLS 41 kb) Additional file 9: T**he primers used in this study.** (XLSX 11 kb)

## Abbreviations

miRNAs: MicroRNAs
sRNAs: Small RNAs
nt: Nucleotide
1-MCP: 1-Methylcyclopropene
ET: Ethylene
CK: Control
MFE: Minimum Free Energy
GO: Gene Ontology
KEGG: Kyoto Encyclopedia of Genes and Genomes
qRT-PCR: Quantitative Reverse Transcription real-time PCR
PLSD: Fisher’s protected Least Significant Difference

## References

[1] Giovannoni JJ (2004). Genetic regulation of fruit development and ripening. Plant Cell.

[2] Seymour GB, Ostergaard L, Chapman NH, Knapp S, Martin C (2013). Fruit development and ripening. Annu Rev Plant Biol.

[3] Gao C, Ju Z, Cao D, Zhai B, Qin G, Zhu H (2015). MicroRNA profiling analysis throughout tomato fruit development and ripening reveals potential regulatory role of RIN on microRNAs accumulation. Plant Biotechnol J.

[4] Bapata VA, Trivedib PK, Ghoshc A, Ganapathic VASTR, Nath P (2010). Ripening of fleshy fruit: molecular insight and the role of ethylene. Biotechnol Adv.

[5] Clendennen S, Kipp P, May G, AK K (1997). The role of ethylene in banana fruit ripening. Biology and biotechnology of the plant hormone ethylene.

[6] Liu X, Shiomi S, Nakatsuka A, Kubo Y, Nakamura R, Inaba A (1999). Characterization of ethylene biosynthesis associated with ripening in banana fruit. Plant Physiol.

[7] Elitzur T, Vrebalov J, Giovannoni JJ, Goldschmidt EE, Friedman H (2010). The regulation of MADS-box gene expression during ripening of banana and their regulatory interaction with ethylene. J Exp Bot.

[8] Mbeguie-A-Mbeguie D, Hubert O, Fils-Lycaon B, Chillet M, Baurens FC (2008). EIN3-like gene expression during fruit ripening of Cavendish banana (*Musa acuminata* cv. *Grande naine*). Physiol Plant.

[9] Roy Choudhury S, Roy S, Nag A, Singh SK, Sengupta DN (2012). Characterization of an AGAMOUS-like MADS box protein, a probable constituent of flowering and fruit ripening regulatory system in banana. PLoS One.

[10] Shan W, Kuang JF, Chen L, Xie H, Peng HH, Xiao YY (2012). Molecular characterization of banana NAC transcription factors and their interactions with ethylene signalling component EIL during fruit ripening. J Exp Bot.

[11] Xiao YY, Chen JY, Kuang JF, Shan W, Xie H, Jiang YM (2013). Banana ethylene response factors are involved in fruit ripening through their interactions with ethylene biosynthesis genes. J Exp Bot.

[12] Ba LJ, Shan W, Xiao YY, Chen JY, Lu WJ, Kuang JF (2014). A ripening-induced transcription factor MaBSD1 interacts with promoters of MaEXP1/2 from banana fruit. Plant Cell Rep.

[13] Ba L-J, Shan W, Kuang J-f, Feng B-h, Xiao Y-y, Lu W-j (2014). The Banana MaLBD (LATERAL ORGAN BOUNDARIES DOMAIN) Transcription Factors Regulate EXPANSIN Expression and Are Involved in Fruit Ripening. Plant Mol Biol Rep.

[14] Cuperus JT, Fahlgren N, Carrington JC (2011). Evolution and functional diversification of MIRNA genes. Plant Cell.

[15] Debat HJ, Ducasse DA (2014). Plant microRNAs: recent advances and future challenges. Plant Mol Biol Rep.

[16] Moxon S, Jing R, Szittya G, Schwach F, Rusholme Pilcher RL, Moulton V (2008). Deep sequencing of tomato short RNAs identifies microRNAs targeting genes involved in fruit ripening. Genome Res.

[17] Zuo J, Zhu B, Fu D, Zhu Y, Ma Y, Chi L (2012). Sculpting the maturation, softening and ethylene pathway: the influences of microRNAs on tomato fruits. BMC Genomics.

[18] Zhang X, Zou Z, Zhang J, Zhang Y, Han Q, Hu T (2011). Over-expression of sly-miR156a in tomato results in multiple vegetative and reproductive trait alterations and partial phenocopy of the sft mutant. FEBS Lett.

[19] Gao Z, Shi T, Luo X, Zhang Z, Zhuang W, Wang L (2012). High-throughput sequencing of small RNAs and analysis of differentially expressed microRNAs associated with pistil development in Japanese apricot. BMC Genomics.

[20] Zhu QH, Helliwell CA (2011). Regulation of flowering time and floral patterning by miR172. J Exp Bot.

[21] Silva GFF, Silva EM, Azevedo MS, Guivin MAC, Ramiro DA, Figueiredo CR (2014). microRNA156-targeted SPL/SBP box transcription factors regulate tomato ovary and fruit development. Plant J.

[22] Liu Y, Wang L, Chen D, Wu X, Huang D, Chen L (2014). Genome-wide comparison of microRNAs and their targeted transcripts among leaf, flower and fruit of sweet orange. BMC Genomics.

[23] Chen W, Kong J, Lai T, Manning K, Wu C, Wang Y (2015). Tuning LeSPL-CNR expression by SlymiR157 affects tomato fruit ripening. Sci Rep.

[24] Wu J, Wang D, Liu Y, Wang L, Qiao X, Zhang S (2014). Identification of miRNAs involved in pear fruit development and quality. BMC Genomics.

[25] Aukerman MJ, Sakai H (2003). Regulation of flowering time and floral organ identity by a MicroRNA and its APETALA2-like target genes. Plant Cell.

[26] Chen X (2004). A microRNA as a translational repressor of APETALA2 in *Arabidopsis* flower development. Science.

[27] Karlova R, van Haarst JC, Maliepaard C, van de Geest H, Bovy AG, Lammers M (2013). Identification of microRNA targets in tomato fruit development using high-throughput sequencing and degradome analysis. J Exp Bot.

[28] D’Hont A, Denoeud F, Aury JM, Baurens FC, Carreel F, Garsmeur O (2012). The banana (*Musa acuminata*) genome and the evolution of monocotyledonous plants. Nature.

[29] Chai J, Feng R, Shi H, Ren M, Zhang Y, Wang J (2015). Bioinformatic identification and expression analysis of banana MicroRNAs and their targets. PLoS One.

[30] Wen JZ, Liao JY, Zheng LL, Xu H, Yang JH, Guan DG (2014). A contig-based strategy for the genome-wide discovery of microRNAs without complete genome resources. PLoS One.

[31] Reid MS, Staby GL (2008). A Brief History of 1-Methylcyclopropene. Hortscience.

[32] Tonutti P, Bonghi C, Ramina A. Modulating effects of ethylene and ethylene inhibitors in the control of fruit ripening. In: Ramina A, Chang C, Giovannoni J, Klee H, Perata P, Woltering E, editors. Advances in Plant Ethylene Research: Proceedings of the 7th International Symposium on the Plant Hormone Ethylene. Dordrecht, The Netherlands: online: Springer; 2007. p. 407–15.

[33] Eyles RP, Williams PH, Ohms SJ, Weiller GF, Ogilvie HA, Djordjevic MA (2013). microRNA profiling of root tissues and root forming explant cultures in *Medicago truncatula*. Planta.

[34] Kong X, Zhang M, Xu X, Li X, Li C, Ding Z (2014). System analysis of microRNAs in the development and aluminium stress responses of the maize root system. Plant Biotechnol J.

[35] Lakhotia N, Joshi G, Bhardwaj AR, Katiyar-Agarwal S, Agarwal M, Jagannath A (2014). Identification and characterization of miRNAome in root, stem, leaf and tuber developmental stages of potato (*Solanum tuberosum* L.) by high-throughput sequencing. BMC Plant Biol.

[36] Song C, Wang C, Zhang C, Korir NK, Yu H, Ma Z (2010). Deep sequencing discovery of novel and conserved microRNAs in trifoliate orange (*Citrus trifoliata*). BMC Genomics.

[37] Kou SJ, Wu XM, Liu Z, Liu YL, Xu Q, Guo WW (2012). Selection and validation of suitable reference genes for miRNA expression normalization by quantitative RT-PCR in citrus somatic embryogenic and adult tissues. Plant Cell Rep.

[38] Rajagopalan R, Vaucheret H, Trejo J, Bartel DP (2006). A diverse and evolutionarily fluid set of microRNAs in *Arabidopsis thaliana*. Genes Dev.

[39] Morin RD, Aksay G, Dolgosheina E, Ebhardt HA, Magrini V, Mardis ER (2008). Comparative analysis of the small RNA transcriptomes of Pinus contorta and *Oryza sativa*. Genome Res.

[40] Yao Y, Guo G, Ni Z, Sunkar R, Du J, Zhu JK (2007). Cloning and characterization of microRNAs from wheat (*Triticum aestivum* L.). Genome Biol.

[41] Pantaleo V, Szittya G, Moxon S, Miozzi L, Moulton V, Dalmay T (2010). Identification of grapevine microRNAs and their targets using high-throughput sequencing and degradome analysis. Plant J.

[42] Qiu D, Pan X, Wilson IW, Li F, Liu M, Teng W (2009). High throughput sequencing technology reveals that the taxoid elicitor methyl jasmonate regulates microRNA expression in Chinese yew (*Taxus chinensis*). Gene.

[43] Thiebaut F, Grativol C, Tanurdzic M, Carnavale-Bottino M, Vieira T, Motta MR (2014). Differential sRNA regulation in leaves and roots of sugarcane under water depletion. PLoS One.

[44] Liu HW, Luo LX, Liang CQ, Jiang N, Liu PF, Li JQ (2015). High-throughput sequencing identifies novel and conserved cucumber (*Cucumis sativus* L.) microRNAs in response to cucumber green mottle mosaic virus infection. PLoS One.

[45] Zhao CZ, Xia H, Frazier TP, Yao YY, Bi YP, Li AQ (2010). Deep sequencing identifies novel and conserved microRNAs in peanuts (*Arachis hypogaea* L.). BMC Plant Biol.

[46] Meyers BC, Axtell MJ, Bartel B, Bartel DP, Baulcombe D, Bowman JL (2008). Criteria for annotation of plant MicroRNAs. Plant Cell.

[47] Yu R, Wang Y, Xu L, Zhu X, Zhang W, Wang R (2015). Transcriptome profiling of root microRNAs reveals novel insights into taproot thickening in radish (*Raphanus sativus* L.). BMC Plant Biol.

[48] Bonnet E, Wuyts J, Fau Rouze P, Fau RP, Van de Peer Y, Van de Peer Y (2004). Evidence that microRNA precursors, unlike other non-coding RNAs, have lower folding free energies than random sequences. Bioinformatics.

[49] Wang JW, Czech B, Weigel D (2009). miR156-regulated SPL transcription factors define an endogenous flowering pathway in *Arabidopsis thaliana*. Cell.

[50] Gandikota M, Birkenbihl RP, Hohmann S, Cardon GH, Saedler H, Huijser P (2007). The miRNA156/157 recognition element in the 3’ UTR of the Arabidopsis SBP box gene SPL3 prevents early flowering by translational inhibition in seedlings. Plant J.

[51] Aharoni A, De Vos CH, Wein M, Sun Z, Greco R, Kroon A (2001). The strawberry FaMYB1 transcription factor suppresses anthocyanin and flavonol accumulation in transgenic tobacco. Plant J.

[52] Tsuji H, Aya K, Ueguchi-Tanaka M, Shimada Y, Nakazono M, Watanabe R (2006). GAMYB controls different sets of genes and is differentially regulated by microRNA in aleurone cells and anthers. Plant J.

[53] Sorin C, Declerck M, Christ A, Blein T, Ma L, Lelandais-Briere C (2014). A miR169 isoform regulates specific NF-YA targets and root architecture in *Arabidopsis*. New Phytol.

[54] Guo HS, Xie Q, Fei JF, Chua NH (2005). MicroRNA directs mRNA cleavage of the transcription factor NAC1 to downregulate auxin signals for arabidopsis lateral root development. Plant Cell.

[55] Gutierrez L, Bussell J, Pacurar D, Schwambach J, Pacurar M, Bellini C (2009). Phenotypic plasticity of adventitious rooting in *Arabidopsis* is controlled by complex regulation of AUXIN RESPONSE FACTOR transcripts and microRNA abundance. Plant Cell.

[56] Kumar R, Sharma AK, Nath P, Bouzayen M, Pech JC, Mattoo AK (2014). Ethylene perception and signaling in ripening fruit. Fruit ripening: physiology, signalling and genomics.

[57] Wang Y, Wang W, Cai J, Zhang Y, Qin G, Tian S (2014). Tomato nuclear proteome reveals the involvement of specific E2 ubiquitin-conjugating enzymes in fruit ripening. Genome Biol.

[58] Kevany BM, Tieman DM, Taylor MG, Cin VD, Klee HJ (2007). Ethylene receptor degradation controls the timing of ripening in tomato fruit. Plant J.

[59] Skottke KR, Yoon GM, Kieber JJ, DeLong A (2011). Protein phosphatase 2A controls ethylene biosynthesis by differentially regulating the turnover of ACC synthase isoforms. PLoS Genet.

[60] Carey AT, Smith DL, Harrison E, Bird CR, Gross KC, Seymour GB (2001). Down-regulation of a ripening-related beta-galactosidase gene (TBG1) in transgenic tomato fruits. J Exp Bot.

[61] Mazzuca S, Spadafora A, Innocenti AM (2006). Cell and tissue localization of β-glucosidase during the ripening of olive fruit (*Olea europae*a) by in situ activity assay. Plant Sci.

[62] J-h S, Dong Y-h, C-l L, Shen Y-y (2014). Transcription and enzymatic analysis of beta-glucosidase VvBG1 in grape berry ripening. Plant Growth Regul.

[63] Hobert O (2008). Gene regulation by transcription factors and microRNAs. Science.

[64] Cherian S, Figueroa CR, Nair H (2014). ‘Movers and shakers’ in the regulation of fruit ripening: a cross-dissection of climacteric versus non-climacteric fruit. J Exp Bot.

[65] Tieman DM, Ciardi JA, Taylor MG, Klee HJ (2001). Members of the tomato LeEIL (EIN3-like) gene family are functionally redundant and regulate ethylene responses throughout plant development. Plant J.

[66] Yokotani N, Nakano R, Imanishi S, Nagata M, Inaba A, Kubo Y (2009). Ripening-associated ethylene biosynthesis in tomato fruit is autocatalytically and developmentally regulated. J Exp Bot.

[67] Kawashima CG, Matthewman CA, Huang S, Lee BR, Yoshimoto N, Koprivova A (2011). Interplay of SLIM1 and miR395 in the regulation of sulfate assimilation in *Arabidopsis*. Plant J.

[68] Wan CY, Wilkins TA (1994). A modified hot borate method significantly enhances the yield of high-quality RNA from cotton (*Gossypium hirsutum* L.). Anal Biochem.

[69] Langmead B, Trapnell C, Pop M, Salzberg SL (2009). Ultrafast and memory-efficient alignment of short DNA sequences to the human genome. Genome Biol.

[70] Wen M, Shen Y, Shi S, Tang T (2012). miREvo: an integrative microRNA evolutionary analysis platform for next-generation sequencing experiments. BMC Bioinformatics.

[71] Friedlander MR, Mackowiak SD, Li N, Chen W, Rajewsky N (2012). miRDeep2 accurately identifies known and hundreds of novel microRNA genes in seven animal clades. Nucleic Acids Res.

[72] Wang L, Feng Z, Wang X, Wang X, Zhang X (2010). DEGseq: an R package for identifying differentially expressed genes from RNA-seq data. Bioinformatics.

[73] Storey JD (2003). The positive false discovery rate: a Bayesian interpretation and the q-value. Ann Statist.

[74] Wu HJ, Ma YK, Chen T, Wang M, Wang XJ (2012). PsRobot: a web-based plant small RNA meta-analysis toolbox. Nucleic Acids Res.

[75] Chen L, Zhong HY, Kuang JF, Li JG, Lu WJ, Chen JY (2011). Validation of reference genes for RT-qPCR studies of gene expression in banana fruit under different experimental conditions. Planta.

[76] Varkonyi-Gasic E, Wu R, Wood M, Walton EF, Hellens RP (2007). Protocol: a highly sensitive RT-PCR method for detection and quantification of microRNAs. Plant Methods.

