# Supplementary material for: Identification of miRNAs involved in fruit ripening in Cavendish bananas by deep sequencing
Source: BMC Genomics. 2015 Oct 13;16:776. doi: 10.1186/s12864-015-1995-1 (PMC4603801; doi:10.1186/s12864-015-1995-1)
Supplement: Additional file 1: — Physiological changes of banana fruit after ethylene or 1-MCP treatment. (DOCX 422 kb) [file 12864_2015_1995_MOESM1_ESM.docx]

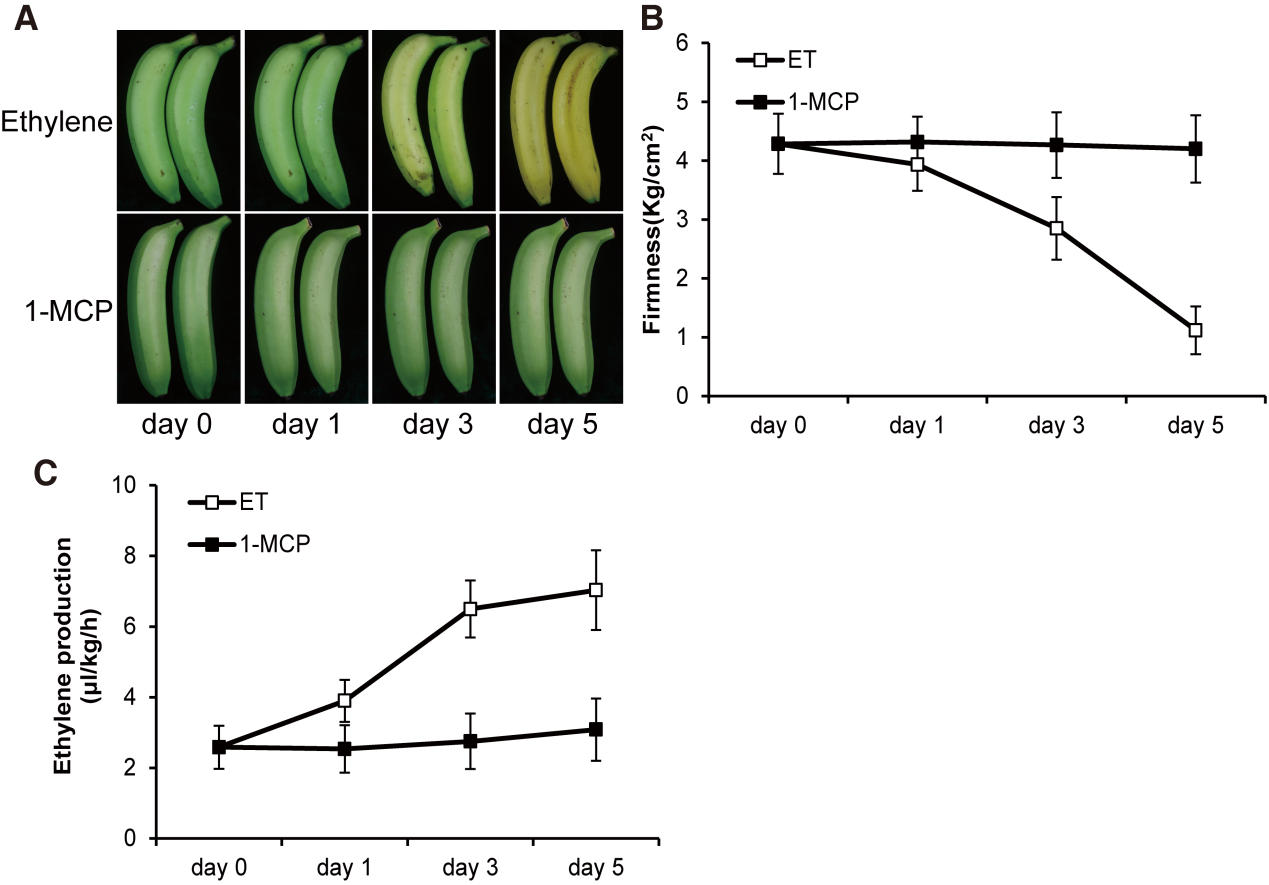


**Additional file 1 physiological changes of banana fruit after ethylene or 1-MCP treatment**

**(A) The photograph of banana fruit after different treatment.** And alll samples were collected for qRT-PCR validation of differentially expressed miRNAs and their target genes. The banana fruit in day 3 after ethylent or 1-MCP treatment and the banana fruit before treatment were collected for sRNA sequencing.

**(B) Firmness, (C) ethylene production of banana fruit after different treatment.** The result is mean value of 6 replicates for firmness, 3 replicates for ethylene production. Bars indicate Standard Error (SE).
