# Supplementary figures and images for: Identification of miRNAs involved in fruit ripening in Cavendish bananas by deep sequencing
Source: BMC Genomics. 2015 Oct 13;16:776. doi: 10.1186/s12864-015-1995-1 (PMC4603801; doi:10.1186/s12864-015-1995-1)

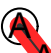

Supplement: Additional file 4: — The secondary structure of an identified novel miRNA precursor Secondary structure of 26 novel miRNA precursor is included. (ZIP 95 kb) [file 12864_2015_1995_MOESM4_ESM.zip › Additional file 4 the second structure of identified novel miRNAs/Mac-nmiR1.pdf]

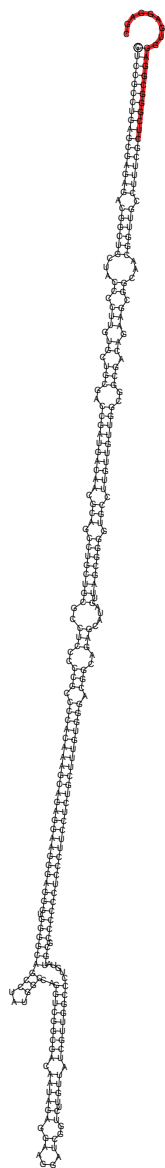

Secondary structure for 'novel\_139'

Supplement: Additional file 4: — The secondary structure of an identified novel miRNA precursor Secondary structure of 26 novel miRNA precursor is included. (ZIP 95 kb) [file 12864_2015_1995_MOESM4_ESM.zip › Additional file 4 the second structure of identified novel miRNAs/Mac-nmiR10.pdf]

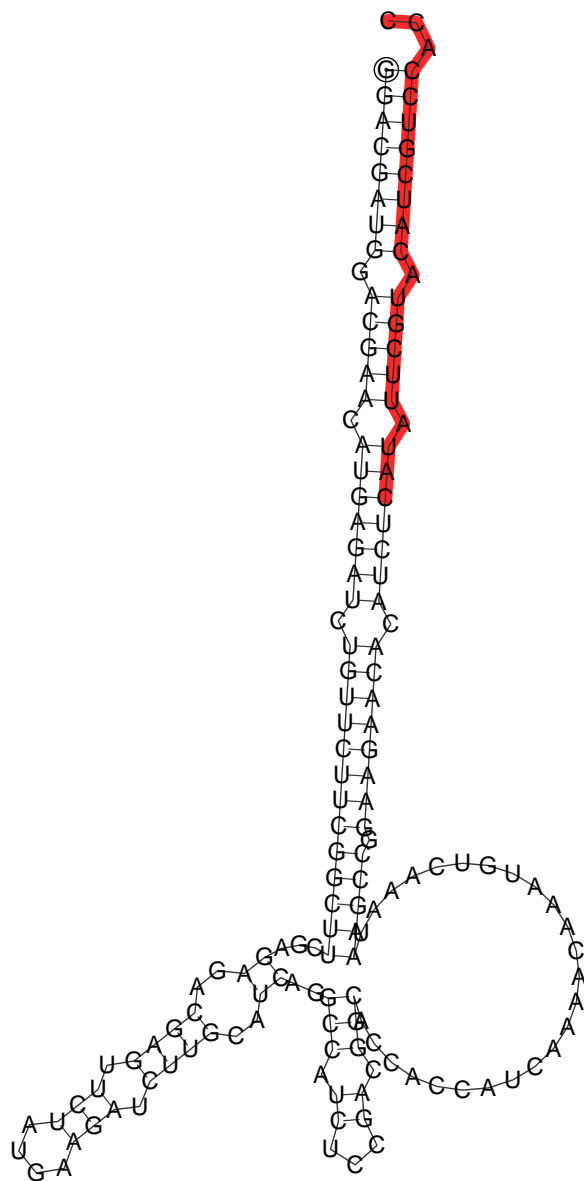

Secondary structure for 'novel\_15'

Supplement: Additional file 4: — The secondary structure of an identified novel miRNA precursor Secondary structure of 26 novel miRNA precursor is included. (ZIP 95 kb) [file 12864_2015_1995_MOESM4_ESM.zip › Additional file 4 the second structure of identified novel miRNAs/Mac-nmiR11-3p.pdf]

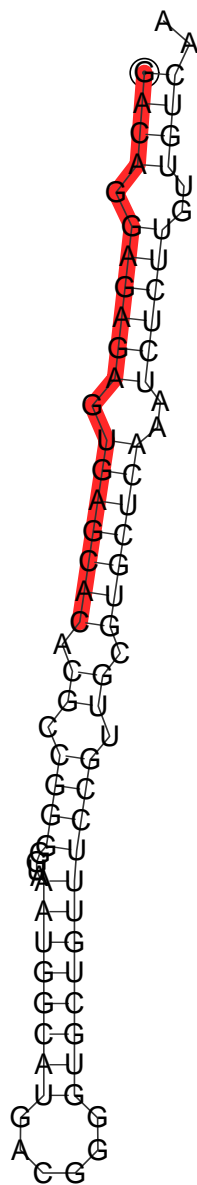

Secondary structure for 'novel\_152'

Supplement: Additional file 4: — The secondary structure of an identified novel miRNA precursor Secondary structure of 26 novel miRNA precursor is included. (ZIP 95 kb) [file 12864_2015_1995_MOESM4_ESM.zip › Additional file 4 the second structure of identified novel miRNAs/Mac-nmiR12.pdf]

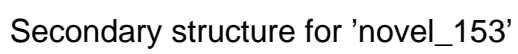

Supplement: Additional file 4: — The secondary structure of an identified novel miRNA precursor Secondary structure of 26 novel miRNA precursor is included. (ZIP 95 kb) [file 12864_2015_1995_MOESM4_ESM.zip › Additional file 4 the second structure of identified novel miRNAs/Mac-nmiR13.pdf]

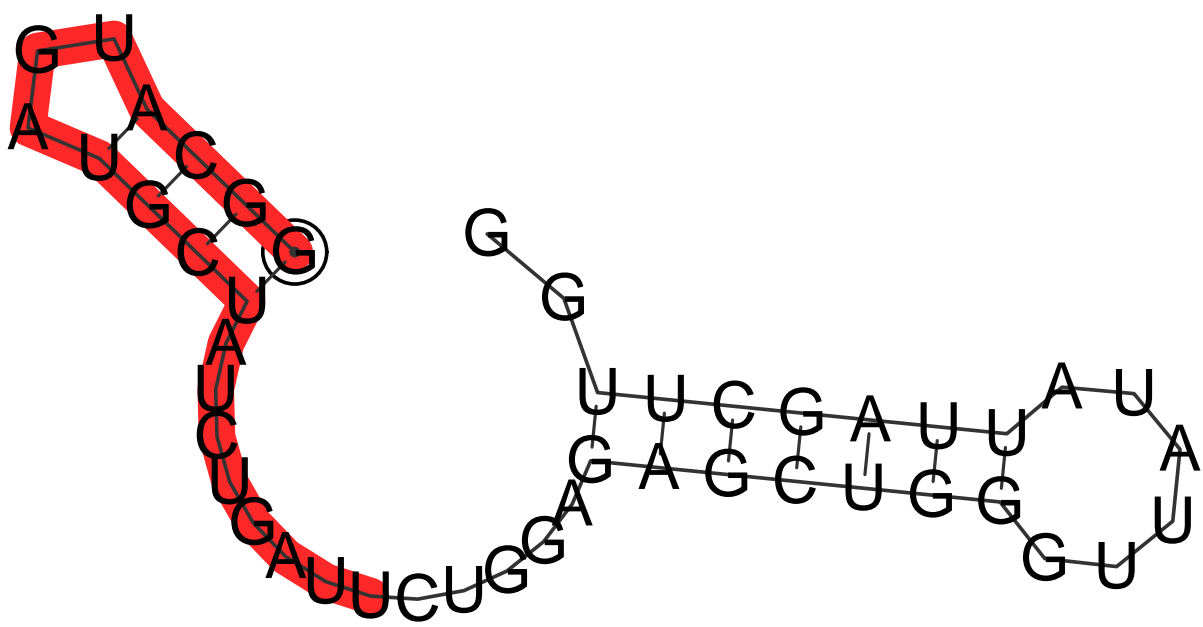

Secondary structure for 'novel\_162'

Supplement: Additional file 4: — The secondary structure of an identified novel miRNA precursor Secondary structure of 26 novel miRNA precursor is included. (ZIP 95 kb) [file 12864_2015_1995_MOESM4_ESM.zip › Additional file 4 the second structure of identified novel miRNAs/Mac-nmiR14.pdf]

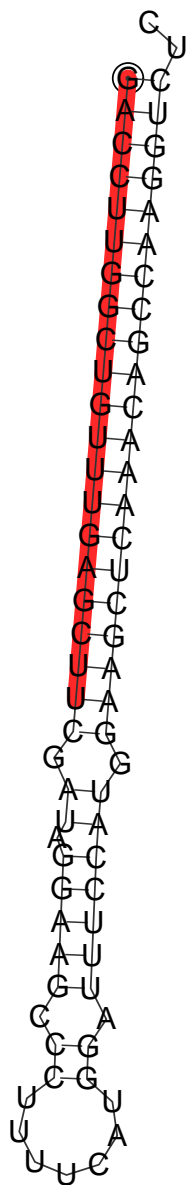

Supplement: Additional file 4: — The secondary structure of an identified novel miRNA precursor Secondary structure of 26 novel miRNA precursor is included. (ZIP 95 kb) [file 12864_2015_1995_MOESM4_ESM.zip › Additional file 4 the second structure of identified novel miRNAs/Mac-nmiR15-5p.pdf]

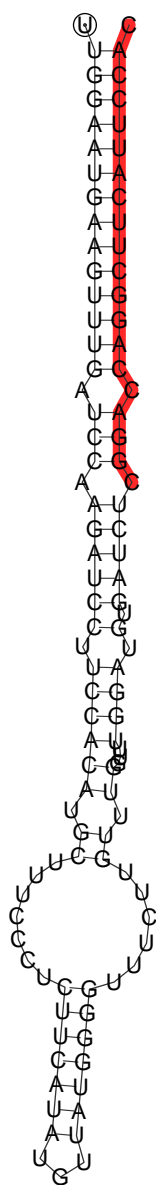

Secondary structure for 'novel\_171'

Supplement: Additional file 4: — The secondary structure of an identified novel miRNA precursor Secondary structure of 26 novel miRNA precursor is included. (ZIP 95 kb) [file 12864_2015_1995_MOESM4_ESM.zip › Additional file 4 the second structure of identified novel miRNAs/Mac-nmiR16.pdf]

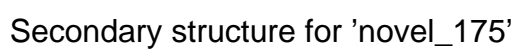

Supplement: Additional file 4: — The secondary structure of an identified novel miRNA precursor Secondary structure of 26 novel miRNA precursor is included. (ZIP 95 kb) [file 12864_2015_1995_MOESM4_ESM.zip › Additional file 4 the second structure of identified novel miRNAs/Mac-nmiR17.pdf]

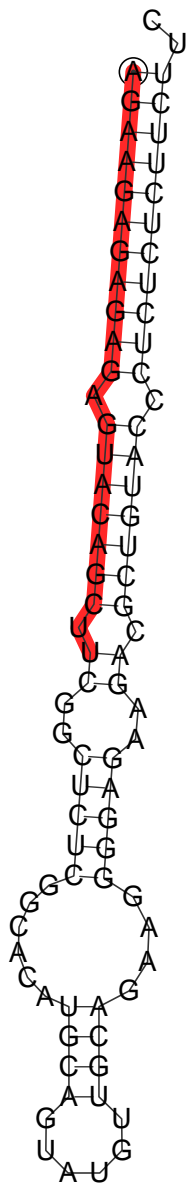

Supplement: Additional file 4: — The secondary structure of an identified novel miRNA precursor Secondary structure of 26 novel miRNA precursor is included. (ZIP 95 kb) [file 12864_2015_1995_MOESM4_ESM.zip › Additional file 4 the second structure of identified novel miRNAs/Mac-nmiR18-5p.pdf]

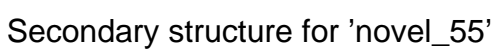

Supplement: Additional file 4: — The secondary structure of an identified novel miRNA precursor Secondary structure of 26 novel miRNA precursor is included. (ZIP 95 kb) [file 12864_2015_1995_MOESM4_ESM.zip › Additional file 4 the second structure of identified novel miRNAs/Mac-nmiR19-5p.pdf]

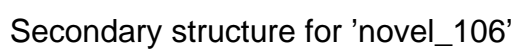

Supplement: Additional file 4: — The secondary structure of an identified novel miRNA precursor Secondary structure of 26 novel miRNA precursor is included. (ZIP 95 kb) [file 12864_2015_1995_MOESM4_ESM.zip › Additional file 4 the second structure of identified novel miRNAs/Mac-nmiR2.pdf]

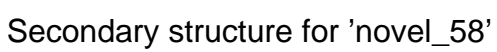

Supplement: Additional file 4: — The secondary structure of an identified novel miRNA precursor Secondary structure of 26 novel miRNA precursor is included. (ZIP 95 kb) [file 12864_2015_1995_MOESM4_ESM.zip › Additional file 4 the second structure of identified novel miRNAs/Mac-nmiR20-5p.pdf]

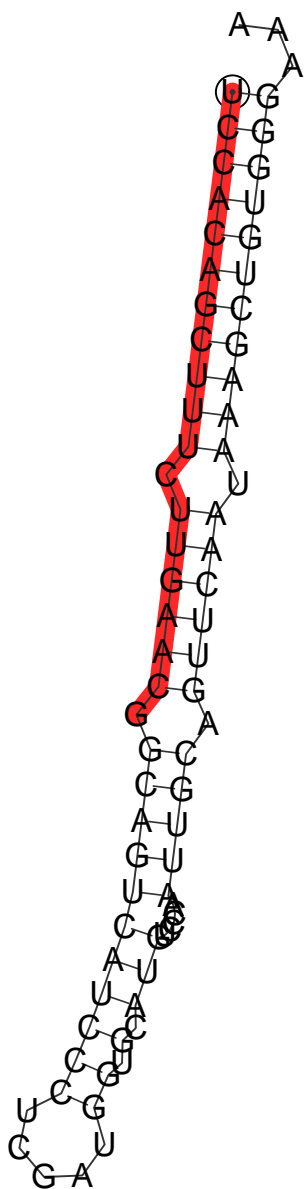

Secondary structure for 'novel\_70'

Supplement: Additional file 4: — The secondary structure of an identified novel miRNA precursor Secondary structure of 26 novel miRNA precursor is included. (ZIP 95 kb) [file 12864_2015_1995_MOESM4_ESM.zip › Additional file 4 the second structure of identified novel miRNAs/Mac-nmiR21.pdf]

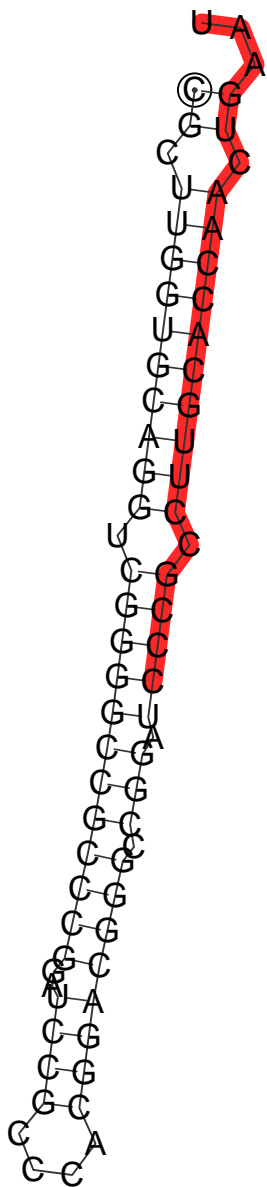

Secondary structure for 'novel\_90'

Supplement: Additional file 4: — The secondary structure of an identified novel miRNA precursor Secondary structure of 26 novel miRNA precursor is included. (ZIP 95 kb) [file 12864_2015_1995_MOESM4_ESM.zip › Additional file 4 the second structure of identified novel miRNAs/Mac-nmiR24-3p.pdf]

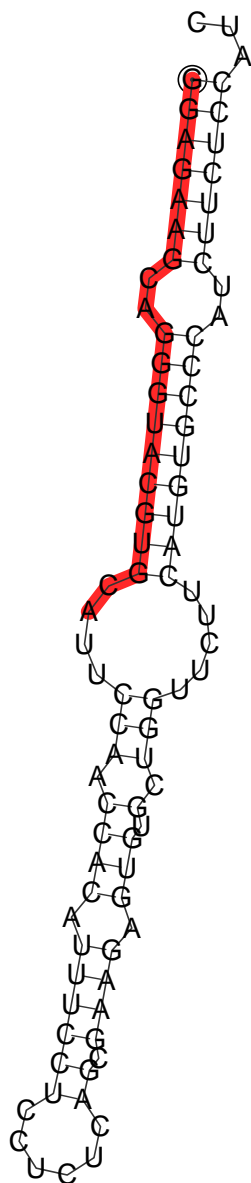

Secondary structure for 'novel\_92'

Supplement: Additional file 4: — The secondary structure of an identified novel miRNA precursor Secondary structure of 26 novel miRNA precursor is included. (ZIP 95 kb) [file 12864_2015_1995_MOESM4_ESM.zip › Additional file 4 the second structure of identified novel miRNAs/Mac-nmiR25.pdf]

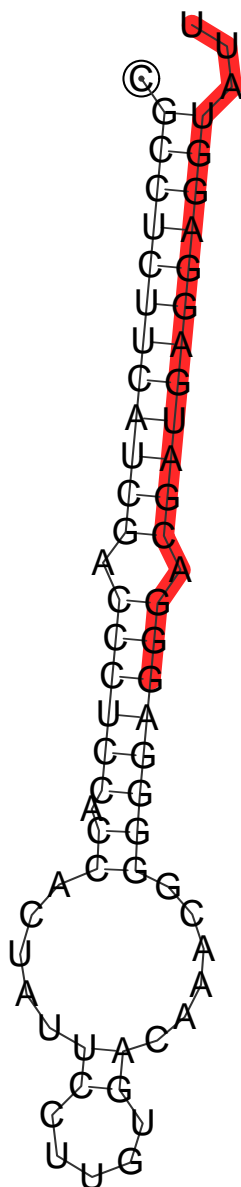

Secondary structure for 'novel\_99'

Supplement: Additional file 4: — The secondary structure of an identified novel miRNA precursor Secondary structure of 26 novel miRNA precursor is included. (ZIP 95 kb) [file 12864_2015_1995_MOESM4_ESM.zip › Additional file 4 the second structure of identified novel miRNAs/Mac-nmiR26-3p.pdf]

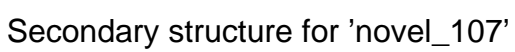

Supplement: Additional file 4: — The secondary structure of an identified novel miRNA precursor Secondary structure of 26 novel miRNA precursor is included. (ZIP 95 kb) [file 12864_2015_1995_MOESM4_ESM.zip › Additional file 4 the second structure of identified novel miRNAs/Mac-nmiR3.pdf]

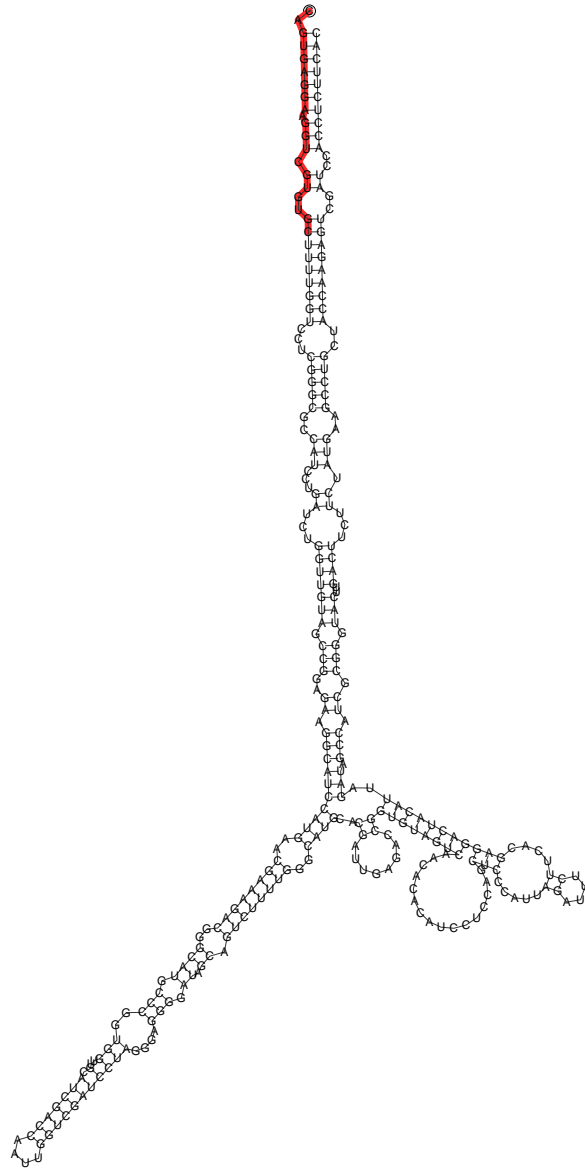

Secondary structure for 'novel\_113'

Supplement: Additional file 4: — The secondary structure of an identified novel miRNA precursor Secondary structure of 26 novel miRNA precursor is included. (ZIP 95 kb) [file 12864_2015_1995_MOESM4_ESM.zip › Additional file 4 the second structure of identified novel miRNAs/Mac-nmiR4.pdf]

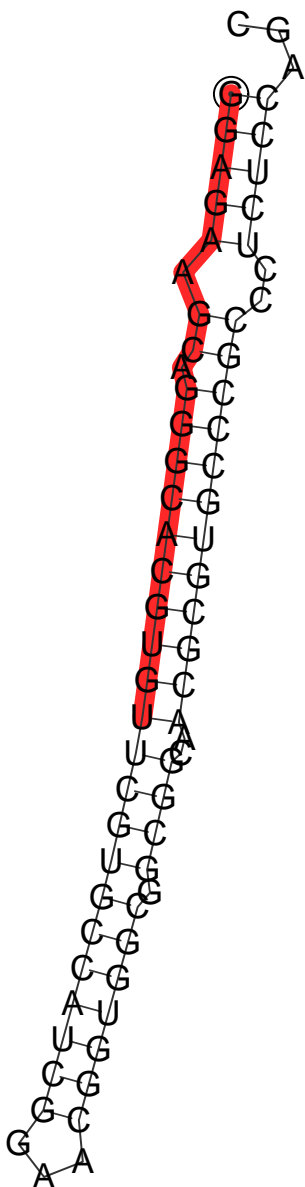

Secondary structure for 'novel\_114'

Supplement: Additional file 4: — The secondary structure of an identified novel miRNA precursor Secondary structure of 26 novel miRNA precursor is included. (ZIP 95 kb) [file 12864_2015_1995_MOESM4_ESM.zip › Additional file 4 the second structure of identified novel miRNAs/Mac-nmiR5.pdf]

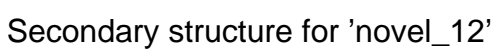

Supplement: Additional file 4: — The secondary structure of an identified novel miRNA precursor Secondary structure of 26 novel miRNA precursor is included. (ZIP 95 kb) [file 12864_2015_1995_MOESM4_ESM.zip › Additional file 4 the second structure of identified novel miRNAs/Mac-nmiR6.pdf]

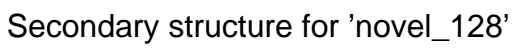

Supplement: Additional file 4: — The secondary structure of an identified novel miRNA precursor Secondary structure of 26 novel miRNA precursor is included. (ZIP 95 kb) [file 12864_2015_1995_MOESM4_ESM.zip › Additional file 4 the second structure of identified novel miRNAs/Mac-nmiR7-5p.pdf]

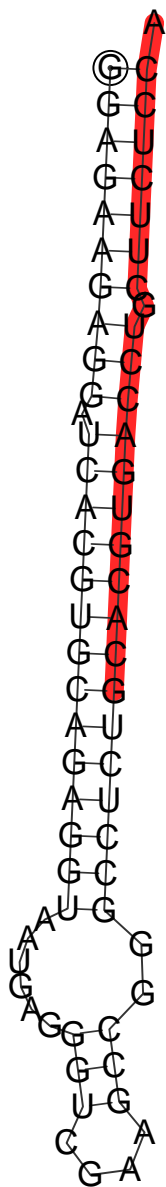

Supplement: Additional file 4: — The secondary structure of an identified novel miRNA precursor Secondary structure of 26 novel miRNA precursor is included. (ZIP 95 kb) [file 12864_2015_1995_MOESM4_ESM.zip › Additional file 4 the second structure of identified novel miRNAs/Mac-nmiR8-3p.pdf]

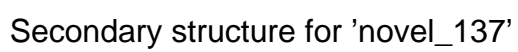

Supplement: Additional file 4: — The secondary structure of an identified novel miRNA precursor Secondary structure of 26 novel miRNA precursor is included. (ZIP 95 kb) [file 12864_2015_1995_MOESM4_ESM.zip › Additional file 4 the second structure of identified novel miRNAs/Mac-nmiR9-3p.pdf]
