# Supplementary material for: Identification of miRNAs involved in fruit ripening in Cavendish bananas by deep sequencing
Source: BMC Genomics. 2015 Oct 13;16:776. doi: 10.1186/s12864-015-1995-1 (PMC4603801; doi:10.1186/s12864-015-1995-1)
Supplement: Additional file 4: — The secondary structure of an identified novel miRNA precursor Secondary structure of 26 novel miRNA precursor is included. (ZIP 95 kb) [file Mac-nmiR22-5p.pdf › Additional file 4 the second structure of identified novel miRNAs/Mac-nmiR22-5p.pdf]

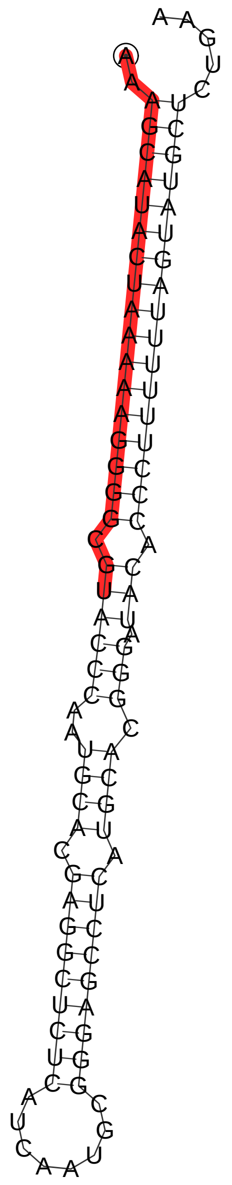

Secondary structure for ’novel_83’
